# Synthetic OCT-A blood vessel maps using fundus images and generative adversarial networks

**DOI:** 10.1038/s41598-023-42062-9

**Published:** 2023-09-15

**Authors:** Ivan Coronado, Samiksha Pachade, Emanuele Trucco, Rania Abdelkhaleq, Juntao Yan, Sergio Salazar-Marioni, Amanda Jagolino-Cole, Mozhdeh Bahrainian, Roomasa Channa, Sunil A. Sheth, Luca Giancardo

**Affiliations:** 1 McWilliams School of Biomedical Informatics, University of Texas Health Science Center at Houston, Houston, TX USA; 2 VAMPIRE project, School of Science and Engineering (Computing), University of Dundee, Dundee, Scotland, UK; 3 McGovern Medical School, University of Texas Health Science Center at Houston, Houston, TX USA; 4 Department of Ophthalmology and Visual Sciences, University of Wisconsin-Madison, Madison, WI USA

**Keywords:** Medical imaging, Computer science, Retina

## Abstract

Vessel segmentation in fundus images permits understanding retinal diseases and computing image-based biomarkers. However, manual vessel segmentation is a time-consuming process. Optical coherence tomography angiography (OCT-A) allows direct, non-invasive estimation of retinal vessels. Unfortunately, compared to fundus images, OCT-A cameras are more expensive, less portable, and have a reduced field of view. We present an automated strategy relying on generative adversarial networks to create vascular maps from fundus images without training using manual vessel segmentation maps. Further post-processing used for standard *en face* OCT-A allows obtaining a vessel segmentation map. We compare our approach to state-of-the-art vessel segmentation algorithms trained on manual vessel segmentation maps and vessel segmentations derived from OCT-A. We evaluate them from an automatic vascular segmentation perspective and as vessel density estimators, i.e., the most common imaging biomarker for OCT-A used in studies. Using OCT-A as a training target over manual vessel delineations yields improved vascular maps for the optic disc area and compares to the best-performing vessel segmentation algorithm in the macular region. This technique could reduce the cost and effort incurred when training vessel segmentation algorithms. To incentivize research in this field, we will make the dataset publicly available to the scientific community.

## Introduction

Fundus cameras enable the assessment of the retina in a non-invasive, radiation-free fashion. Both portable and inexpensive, they are frequently used in large population screening studies providing the most common retina imaging modality to date. While multiple retinal structures (e.g., various retinal lesions, optic disc, fovea, and choroidal vessels) are visible in fundus photographs, retinal blood vessels are some of the most studied. Vessels are useful in the diagnosis and prognosis of retinal diseases^1^ and important in the identification of image-based biomarkers associated with conditions like diabetes, glaucoma, and hypertension^2^. Most image-based vascular biomarkers (e.g., fractal dimension, tortuosity, artery-to-vein ratio) rely crucially on a precise segmentation of the retinal vessels. However, manually creating an accurate vessel segmentation is a tedious and time-consuming process unfeasible in many applications, such as disease screening, risk factor computation, or imaging biomarker research on large datasets. According to the Wisconsin Reading Center, a segmentation map of all vessels in a 45° fundus image takes between 30 and 45 min for good quality images and up to an hour for images of poor quality. Even when segmenting a subset of vessels, limited to the optic nerve, and using a semi-automatic approach such as the Integrative Vessel Analysis (IVAN, University of Wisconsin, Madison) software (Wong et al. 2004), it takes about 15 min per image.

Automatic vessel segmentation approaches have been an active area of research for at least 20 years^3^. The best results have been achieved by supervised techniques implementing modifications of fully convolutional neural networks (FCNNs)^4, 5^. When evaluated on publicly available datasets, these approaches have attained performance remarkably close to that of manual readers^3, 6, 7^. However, due to the difficulty of creating manual vessel segmentations, training and validation of these algorithms rely on a limited number of samples representing only a fraction of the morphological variations of the retinal vasculature, especially for non-healthy retinas. Furthermore, manual segmentation of capillary plexi, i.e., small blood vessels, is particularly hard from fundus images as they are not always visible.

Optical coherence tomography angiography (OCT-A) is a relatively novel image modality visualizing blood flow in retinal vessels and microcapillary plexi not clearly visible on fundus images. Near-infrared light permits OCT-A to discriminate static tissues from blood flow, hence allowing blood flow to act as a contrast agent to create vascular maps. Contrary to fluorescein angiography, OCT-A does not require the injection of invasive contrast and visualizes capillary structures more precisely. *En face* images visualize individual vascular layers at different depths segmented from the OCT-A volume scans^8–10^. These images allow for a much better visualization of microcapillary plexi than fundus images.

The most common image biomarker computed from *en face* OCT-A is the local vessel density around the macula and the optic disc^8, 11^. This computation does not require supervised machine learning relying on manual segmentations. However, current commercial OCT-A cameras have limitations: the complex optics of OCT-A cameras make them more expensive, less portable, and with a reduced field of view (FOV) compared to fundus cameras. Therefore, it is not always feasible to use OCT-A in population-based studies or low-resource settings.

In this work, we propose a novel strategy, synthetic OCT-A, to create improved vessel maps estimated from fundus camera images. Instead of training a fully convolutional neural network (FCNN) to map a fundus image to a manually segmented vasculature, we train a FCNN to map a fundus image to an *en face* OCT-A image. We use a custom conditional generative adversarial network (cGAN): our model consists of a generator synthesizing *en face* OCT-A images from corresponding areas in fundus photographs and a discriminator judging the resemblance of the synthesized images with respect to the real *en face* OCT-A images. We evaluate the resulting synthetic OCT-A in two sets of experiments. First, we compute local vessel densities around the macula and the optic disc, which are the de facto standard measurements taken from *en face* OCT-A. Second, we perform a pixel-by-pixel analysis to evaluate the vessel segmentation quality of the synthetic OCT-A. We compare our approach with two FCNN-based vessel segmentation algorithms^4, 5^ currently reporting some of the best vessel segmentation performance on public datasets. Unlike our method, which avoids the need for expert manual vessel delineation, these approaches were pre-trained using a large number of manual vessel maps. Additionally, we evaluate one of these algorithms on retinal vessel segmentation after training it using binary vessel maps captured from OCT-A. Our results demonstrate that it is beneficial to train vessel segmentation methods on the vasculature observed in OCT-A, as manual segmentation of vessels might not correctly capture vascular morphology. Our method is an alternative to manual vessel segmentation, enabling the creation of datasets of fundus images and vascular labels.

Our contributions are as follows:

1. To our best knowledge, we propose the first method to generate synthetic OCT-A *en face* images from fundus images alone without requiring manual vessel segmentation maps.
2. We can generate a full 45° synthetic OCT-A *en face* image from a 45° fundus photograph, even if trained with a smaller FOV.
3. To our best knowledge, we present the first retinal dataset integrating fundus photographs and aligned OCT-A images and make it publicly available for the scientific community.

## Previous work

Recent retinal vessel segmentation algorithms typically leverage representation learning algorithms, more specifically fully convolutional neural networks (FCCNs). One such network is U-Net, a FCCN widely used in biomedical imaging^12^. U-Net is composed of an encoding path and a decoding path. The encoding path has convolutional layers with different number of filters to learn complex image features at different scales. The decoding path considers deconvolutional layers to reconstruct a segmentation map at its original resolution. Skip connections are included between the two paths to facilitate the feature learning process and alleviate the vanishing gradient problem. Since its introduction, researchers have improved and modified this standard model to address the specific needs of different imaging problems. Recent work in retinal image segmentation considers distinct U-Net versions addressing the challenges of automatically delineating retinal blood vessels. Some U-Net variations are worth mentioning in this context.

State-of-the-art methodologies to conduct semantic segmentation often consider an attention mechanism that directs the model to focus on relevant aspects of data. For example, Guo et al. proposed the Spatial Attention U-Net (SA-UNet), which integrates DropBlock^13^ and batch normalization (BN) to prevent overfitting on training data^4^. Spatial attention allows the model to enhance important features (i.e., vasculature) and suppress unimportant features. The FANet model, proposed by Li et al. uses a dual attention block, which utilizes horizontal and vertical pooling operations to produce an attention map for long-range contextual information aggregation^14^. AG-Net, proposed by Zhang et al. considers a multi-scale, multi-label segmentation network that has an attention-guided filter that replaces skip connections and upsampling layers^15^. Other relevant approaches have investigated the use of multiple networks into a single model to better process image features by creating refined feature maps. The IterNet model, proposed by Li et al. joins multiple iterations of a mini U-Net creating a deeper model that learns to fix a large number of false vessel patterns through optimization^5^. Weight sharing and skip connections allow the model to avoid overfitting and produce reliable vessel segmentations^5^. GLUE by Lian et al.^16^ combines local and global information by using a Weighted U-Net and a Weighted Residual U-Net in sequence. This approach uses patches from the fundus images, which receive specific global or local pre-processing for each sub-network to process global or local features independently. Khanal and Estrada^17^ introduced a segmentation pipeline using two U-Nets, the first creating vessel likelihood maps and the second classifying ambiguous pixels in selected patches across the images.

Related studies have explored the synthesis of phantom images from different modalities, including OCT-A and fluorescein angiography (FA). A study exploring the synthesis of FA from fundus images demonstrated that a cGAN model can capture complex morphological information of the vasculature and produce a synthetic FA image with high resemblance to the original FA^18^. However, the authors’ intent differs from what is proposed in this work as they aimed to generate a fundus-to-FA transformation leading to images that are perceptually hard to distinguish from the original images. Instead, our aim is to generate vessel representations equivalent or better than those achieved by standard vessel segmentation algorithms without using manually segmented vasculature maps. OCT-A images allow for a more precise visualization of small capillaries than FA; therefore, fundus-to-*en face* OCT-A transformations will permit generating improved vessel representations. Additionally, acquiring OCT-A images does not require injecting a contrast medium, thus facilitating the creation of relatively large datasets to train deep learning approaches. Another recent cGAN model applied to retina images is by Lee et al.^19^ who created retinal blood flow maps by constructing a synthetic OCT-A image from its OCT counterpart using a cGAN. Their results showed that an approximation to the retinal blood flow could be estimated from OCT structural features, again a different aim from that of our work.

Further work exploring the transformation of fundus images to OCT-A is still needed. Such a transformation would be particularly valuable as it could exploit the advantages of each imaging modality. Fundus photography is widely available and easily performed. OCT-A reveals precise and quantitative information about vascular structures and blood perfusion with much higher precision than standard fundus images.

## Methods

### Materials

The study was performed in accordance with the guidelines from the Helsinki Declaration and was approved by the UTHealth IRB with protocol HSC-MS-19-0352. At the time of writing, the study enrolled a total of 112 patients. Patient statistics can be observed in Table 1.

**Table 1:** Patient demographics.

Variable	
Number of patients	112
Age: mean, years (SD)	45.3 (17.2)
Gender (% male)	48.20%
Race	20.5% Asian, 22.3% Black,
	54.5% White,
	2.7% Other
Ethnicity (% Hispanic)	17.80%
Number of patients without retinal pathologies	99 (88.39%)

Trained graduate research assistants acquired the images after patient stabilization, clinical evaluation, and having obtained informed consent. The images were transferred to a HIPAA compliant cloud storage. Image acquisition was conducted using the OptoVue iCam fundus camera and OptoVue Avanti OCT camera with OCT-A reconstruction software AngioVue. The iCam camera acquires fundus images with a resolution of 2592 × 1944 pixels and FOV of 45°. The Avanti camera can capture OCT-A volumes with FOV 4.5 × 4.5 mm for disc and, 6 × 6 mm and 3 × 3 mm for macula. Both optic disc-centered and macula-centered images were collected. The dataset contains 2D superficial (Inner limiting membrane (ILM) to inner plexiform membrane (IPL)) *en face* projections of the OCT-A images. The original resolution for the *en face* images was 400 × 400 pixels for optic disc and 6 × 6 mm macula; 3 × 3 mm macula images had a resolution of 304 × 304 pixels.

Image alignments were conducted to match local features from OCT-A optic disc and macula with corresponding regions in the fundus images. This process used our custom software visualizing OCT-A as foreground and fundus images in the background. An operator conducted Euclidian transformations (resizing, rotation, and translation) on the OCT-A images to match the vascular patterns observed in the fundus images. In cases where distortion effects limited the operator from conducting an exact alignment, registration of large vessels was prioritized.

The Wisconsin Reading Center at University of Wisconsin-Madison, masked to patients’ characteristics, graded the fundus and OCT-A images for quality and any abnormalities. Quality control involved checking for clarity, focus, lighting, and noise in the images. Additionally, artifacts unique to OCT-A images were evaluated, including motion artifacts, projection artifacts, blink lines, and banding. These quality assessments resulted in a score for each image based on a 3-point scale, with 0 representing high quality (i.e., grading can be performed with high confidence) and 2 poor quality (inadequate for grading). Only fundus images scored 0 or 1 and OCT-A images scored 0 were included in the experiments. The final image count was 104 fundus and 185 *en face* OCT-A images.

### Preprocessing

Preprocessing steps were performed on the fundus and OCT-A images to improve model optimization and convergence during training, match the morphological information between modalities, and mitigate potential errors in the generation of synthetic OCT-A.

First, a background mask from each fundus photo was identified from intensity values to eliminate noisy pixels surrounding the fundus and OCT-A areas not observed in the fundus images after alignment. Second, signal intensities were normalized to a -1 to 1 range for both modalities. Finally, the optic disc and macular areas observable in OCT-A were cropped from the fundus images to obtain crops with similar FOV as the OCT-A. No modifications were performed on the modalities’ channels; therefore, retaining the RGB channels from the fundus images and the single channel of the OCT-A.

### Synthetic OCT-A generation

**Figure 1:**
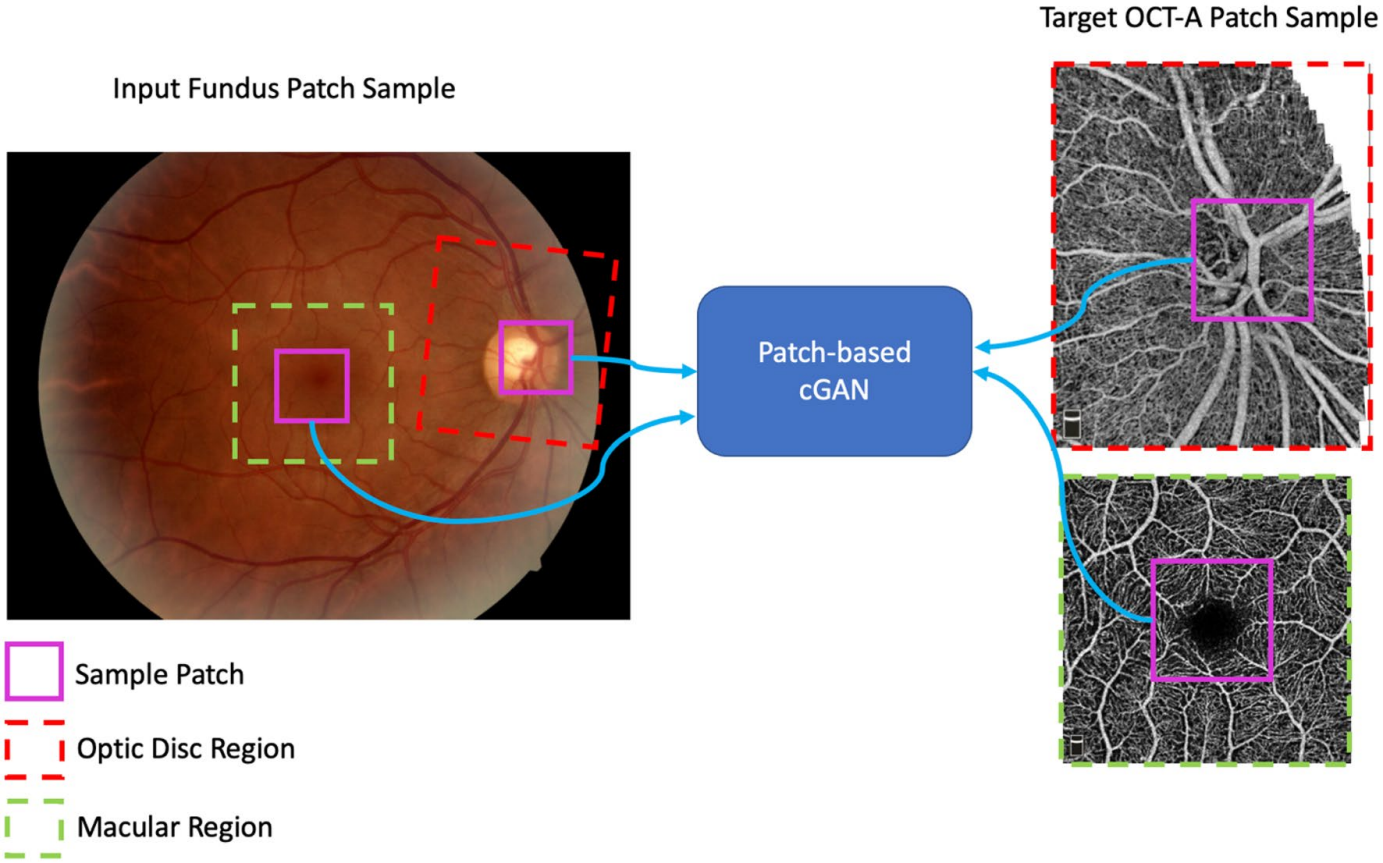
Overview of the Conditional Generative Adversarial Network (cGAN) Patch-based Training. The GAN model represents both the discriminator and generator used in our approach. Red squares represent optic disc regions and green squares enclose the macular regions. Magenta squares represent the patches sampled from the images to train and evaluate the model.

Our method to synthesize OCT-A images is based on cGANs, which have been consistently used in image-to-image transformation tasks^20^. Our model includes two sub-networks: a generator and a discriminator. The networks are set to compete against each other in a minimax game. The generator is tasked with creating realistic *en face* OCT-A samples from a modeled data distribution conditioned by patches extracted from fundus images; the discriminator is set to distinguish between real and synthetic OCT-A patch samples. Our approach follows a similar optimization strategy as conducted in the training of the pix2pix model; however, it differs in the design of the generator network as we utilize a different architectural backbone^21^. Figure 1 shows a depiction of how our patch-based model is optimized to transform an input fundus photo into an *en face* OCT-A image. Red and green squares represent the sampled optic disc and macular regions, respectively.

The magenta squares represent patches sampled within the OCT-A disc and macula images and their counterparts extracted from the fundus image. These patches are used as input and target for the GAN model to learn mappings between the modalities.

**Figure 2:**
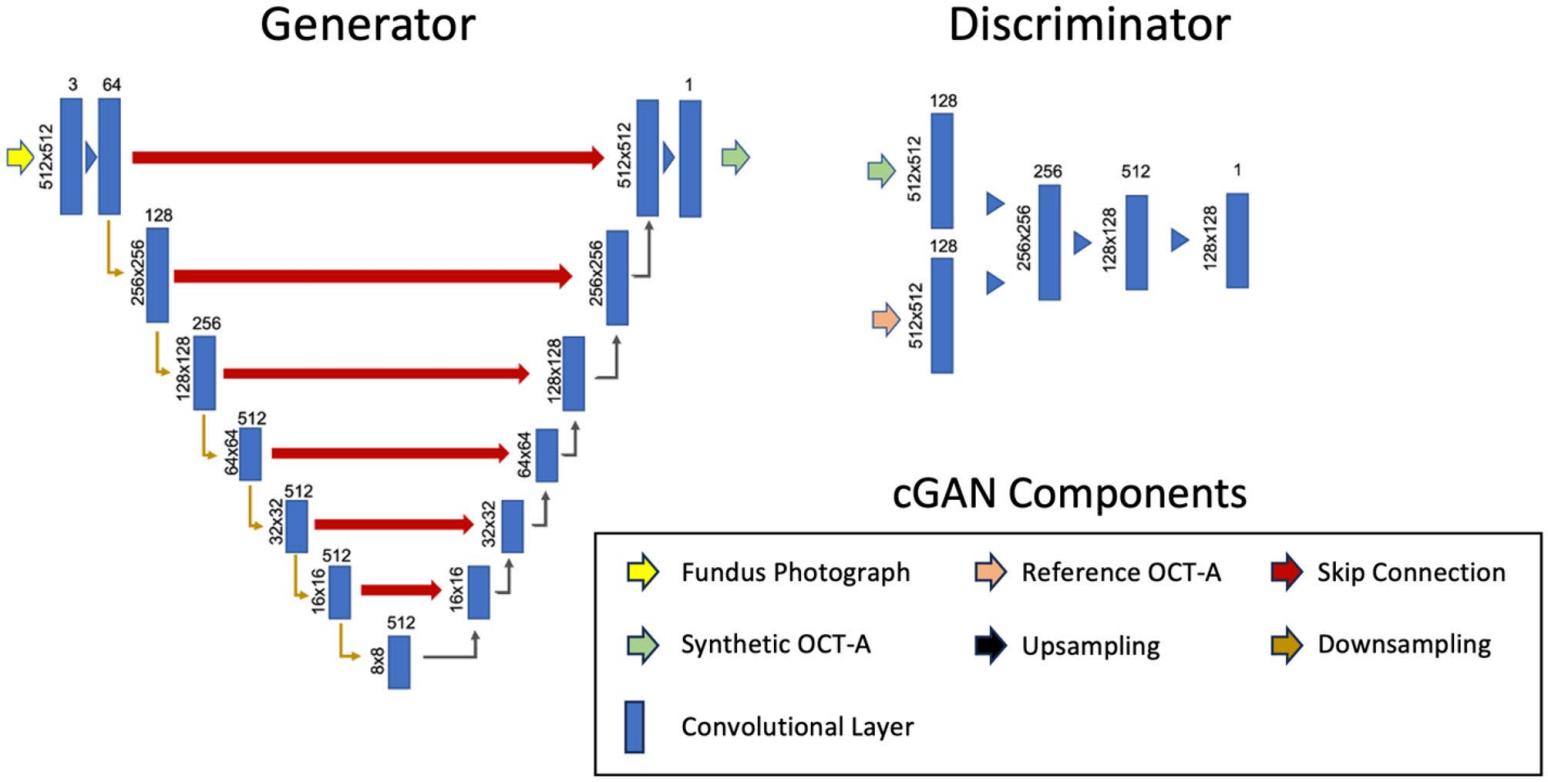
cGAN model architecture. The generator network used is shown on the left and the discriminator network on the right. Vertically oriented values at the left of each convolutional layer rectangle represent the resolution of the feature map, values at the top of each rectangle represent the number of filters/channels for a given layer.

The model’s generator follows the U-Net architecture with encoding and decoding paths mapping features between modalities. The network includes skip connections to preserve features that would otherwise be lost in a pure encoder-decoder model. The discriminator follows a convolutional neural network architecture with convolutional and max-pooling layers encoding image information into a final class vector. Figure 2 shows the overall model architecture.

Successive convolutional layers break down an input fundus photo into features maps of lower resolution. Corresponding deconvolutional layers transform the feature maps back into the original resolution, creating the synthetic version of the OCT-A image. Our deep generator permits localizing large vessels within the images, which are complemented with smaller vasculature added in subsequent layers. Regarding the model training, optimization was conducted using Adam with equal learning rates (2e−4) for both sub-networks. The loss function has two components: the mean squared error between synthetic and real OCT-A samples (1) and the adversarial loss between sub-networks (2). The generator network (G) minimizes the adversarial loss by synthesizing OCT-A images (G(x)) that the discriminator (D) cannot distinguish from real OCT-A samples. The discriminator maximizes the adversarial loss by correctly distinguishing synthetic samples coming from the generator and real OCT-A samples. In the overall model loss (3), the mean squared error is multiplied by a factor λ = 100 to control for artifacts created by the adversarial loss.

1 $$L_{L1}(G) = E_{x,y,z}[||y - G(x)||]$$

2 $$L_{ADV}(G, D) = E_{x,y}[log D(x, y)] + E_{x,y}[log 1 - D(x, G(x)]$$

3 $$L_{GAN} =argminmaxL_{ADV}(G,D)+\lambda L_{L1}(G)$$

where x is the input image, i.e., a local patch in the fundus image, and y is the target image, i.e., the corresponding local patch of the enface OCT-A image. The train/validation/test split was done on a patient basis to avoid any data leakage due to having retinas of the same patient in two splits. We used 25 subjects for training, 5 for validation and the remaining 82 subjects for testing. Using only 25 subjects for training allowed us to roughly match the number of images available in common datasets for vessel segmentation.

At each epoch, 20 patches were randomly sampled from training data. For each OCT-A patch, a complementary fundus photo crop extracted from the macular or optic disc region was used. The patch resolution was set to 512 × 512 × 3. Overall, at each epoch, the model was trained with 120 pairs of patches and validated with 9 pairs of patches. The model was trained for 1000 epochs to learn representations capturing the information from the OCT-A and the fundus images. To avoid overfitting, the weights for the model version that reached the lowest GAN loss were retained. Reflections and rotations were used to augment the data.

Creating a test set required generating synthetic OCT-A images of the macula and optic disc that could be evaluated against the original OCT-A images. To create the set of synthetic OCT-A images, patches were extracted from the 45° FOV fundus photo and processed using the model. A sliding-window approach was followed by sampling patches every 8 pixels along the X axis; when completed, a step of 8 pixels was taken along the Y axis. The process was repeated until the sliding window reached the end of the image. Values from overlapping patches were averaged. Finally, the resulting images were cropped at the macular and optic disc regions matching the FOV observed in the 3 mm and 6 mm macular and 4.5 mm optic disc OCT-A in the ground truth. In other words, only the areas enclosed in green in Fig. 6 are used to train the algorithm.

### SA-UNet and IterNet

Two vessel segmentation methodologies based on convolutional neural networks, SA-UNet^4^and IterNet^5^, were used for comparison. Both methods report some of the best segmentation performance on publicly available retinal datasets (CHASE-DB1^22^ and STARE^23^).

SA-UNet is a model aimed at alleviating the need for thousands of image samples by means of a spatial attention module that refines image features adaptively. The model makes use of DropBlock^13^ and batch normalization to accelerate convergence. Additionally, the architecture has a reduced number of layers compared to the original U-Net addressing the possibility of overfitting training data. We employed a version of SA-UNet provided by the authors, it was trained using data from CHASE-DB1 for a total of 28 images with full 30°/45° FOV.

IterNet employs iterations of mini U-Nets to process and learn the key features of retinal vessels. This approach is advantageous as subsequent networks can learn to fix systematic errors in the vasculature segmentation; additionally, a deeper model allows learning more vasculature patterns showing in retinal images. We used a version of the model provided by the authors; weights were trained using data from DRIVE, CHASE-DB1, and STARE for a total of 56 images with full 30°/45° FOV. Note that a 30°/45° FOV in a fundus image leads to significantly higher number of patches than a single *en face* OCT-A image (see Fig. 1).

In addition, we trained a separate instance of IterNet to segment OCT-A vessels from fundus images. We trained this model using the fundus images and aligned vessel masks segmented from OCT-A. We refer to this method as ‘IterNet w/OCT-A’ for simplicity. The training procedure follows that of the synthetic OCT-A, having patches from the macular and optic disc regions in the fundus photos as input and vessel segmentations obtained from the OCT-A images as target. The patch sampling strategy and data augmentation is the same as for the synthetic OCT-A, given that the images resolution and number of channels remain unchanged. As for the loss function, binary cross entropy measuring the error between the model’s outputs and the ground truth binary masks was used. The model was optimized using Adam with learning rate equal to 1e−3. The model was trained for 1000 epochs; the weights that yielded lowest validation loss were retained.

### Evaluation

Among the methods considered in this work, the synthetic OCT-A is the only one trained with the raw OCT-A images. Therefore, we computed image synthesis metrics (mean absolute error (MAE) and structural similarity index (SSIM)) on the synthetic OCT-A images with respect to the OCT-A ground truth. For the test dataset, the MAE was 40.78 (SD = 3.53) and the SSIM was 0.08 (SD = 0.04).

Further experiments evaluated four distinct vessel segmentation methods. Two of them relying on manual vessels segmentation from fundus images (IterNet and SA-UNet), and the other two relying on vessels delineated from OCT-A using a vessel segmentation algorithm (Synthetic OCT-A Segmented and IterNet w/ OCT-A).

Estimating the vessel density at the optic disc and macular regions is a standard process in the quantification of vascular morphology in OCT-A data^8, 24, 25^. We used this assessment to evaluate how well the segmentations from the different methods approximated the ground truth OCT-A grade vessels based on a biomarker (density) computed from them. We computed precision-recall curves and Areas Under the Curve (AUCs) to assess segmentation performance at the pixel level.

In our evaluation, we considered versions with vessels segmented from both ground truth and synthetic OCT-A to highlight only the pixels belonging to blood vessels. To segment the vessels from OCT-A images, we used the OCT-A vessel segmentation methodology proposed by Ma et al.^26^. The algorithm uses OCTA-Net, a model encompassing different stages of neural networks processing coarse and fine features in *en face* OCT-A images to classify pixels into vessel and non-vessel. The model was trained on 229 OCT-A images each with a corresponding manual vessel delineation provided by an ophthalmologist. The ROSE dataset is a fully independent dataset to our own, provided by the authors^26^.

We first computed Pearson and Spearman correlations for the vessel density measures; specifically, we explored the correlation of vessel density for the optic disc and macula between synthetic and real OCT-A images with the quadrants defined by the Early Treatment Diabetic Retinopathy Study (ETDRS) grid. This grid breaks down the images into four quadrants corresponding to nasal, temporal, superior, and inferior regions. Our experiments used an ETDRS grid with an outer diameter of 3 mm and inner diameter of 1 mm.

Software in commercial cameras employ similar strategies for the extraction of vasculature density measures (sometimes without any segmentation) by not relying on manual annotations, given that if the image is correctly acquired, any pixel in the *en face* OCT-A is a representation of blood perfusion. While vascular density can be computed from non-binarized, raw OCT-A *en face* images, we did not conduct this experiment as the synthetic OCT-A would have had an unfair advantage over the vessel segmentation approaches trained on the binarized vasculature.

In addition, using the target OCT-A pixel-level labels and the probability scores from the different models, we computed precision-recall (PR) curves for macular and optic disc regions. We also provide visualizations for the segmentations produced by both proposed and established methods considered.

### Results

We compare the proposed method using a cGAN and the state-of-the-art vessel segmentation techniques. Tables 2 and 3 show Pearson and Spearman correlation results for the vessel density measures from the real OCT-A to the corresponding measures in the synthetic OCT-A, the IterNet, the IterNet w/ OCT-A, and the SA-UNet vessel segmentations. Significant correlations for the quadrants measured in the macular region are observed for both synthetic OCT-A and the IterNet segmentation. IterNet shows slightly better performance. For the optic disc, synthetic OCT-A and the IterNet w/ OCT-A models achieve the best performance, with significant correlation in all ETDRS quadrants as opposed to the other methods, for which measures do not correlate.

**Table 2:** Optic Disc Vessel Density Correlation between Real OCT-A Images and Vessel Segmentation Methods.

Disc								
	Synthetic OCT-A		IterNet		SA-UNet		IterNet w/ OCT-A	
	Pearson	Spearman	Pearson	Spearman	Pearson	Spearman	Pearson	Spearman
Superior	0.364*	0.372*	− 0.108	− 0.093	− 0.02	− 0.021	0.448*	0.438*
Temporal	0.375*	0.353*	0.175	0.17	0.158	0.168	0.294*	0.333*
Nasal	0.401*	0.371*	0.158	0.159	0.101	0.194	0.583*	0.513*
Fovea	0.334*	0.300*	− 0.014	− 0.058	0.025	− 0.002	0.380*	0.417*
Inferior	0.422*	0.476*	0.14	0.111	− 0.003	0.017	0.534*	0.527*

*denotes significant correlation *p* < 0.05.

**Table 3:** Macula Vessel Density Correlation between Real OCT-A Images and Vessel Segmentation Methods.

Macula								
	Synthetic OCT-A		IterNet		SA-UNet		IterNet w/ OCT-A	
	Pearson	Spearman	Pearson	Spearman	Pearson	Spearman	Pearson	Spearman
Superior	0.154	0.063	0.074	0.062	0.157	0.212	0.192	0.247*
Temporal	0.310*	0.286*	*0.194	0.199*	− 0.007	0.071	0.401*	0.381*
Nasal	0.206	0.121	0.253*	0.177*	− 0.1	− 0.058	0.383*	0.336*
Fovea	− 0.147	0.003	− 0.177	− 0.261	− 0.061	− 0.066	0.258*	0.363*
Inferior	0.331*	0.317*	0.293*	0.285*	0.001	0.088	0.345*	0.347*

*denotes significant correlation *p* < 0.05.

**Figure 3:**
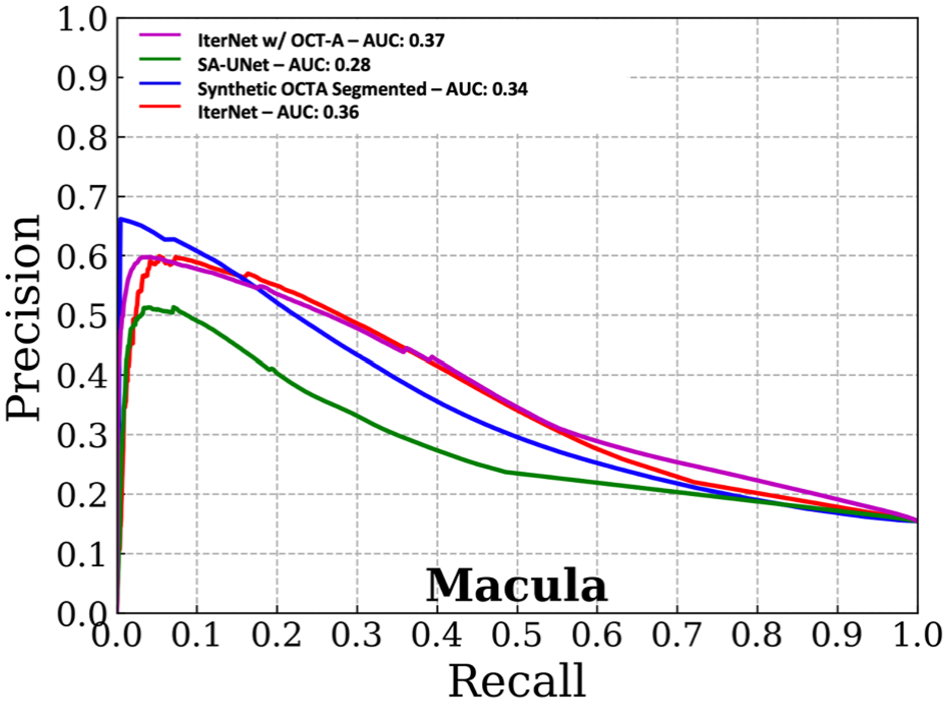
Pixel-wise comparison of our synthetic OCT-A and the two state-of-the-art vessel segmentation algorithms against a binarized OCT-A vasculature on the Macula 3 × 3 mm FOV (test set).

**Figure 4:**
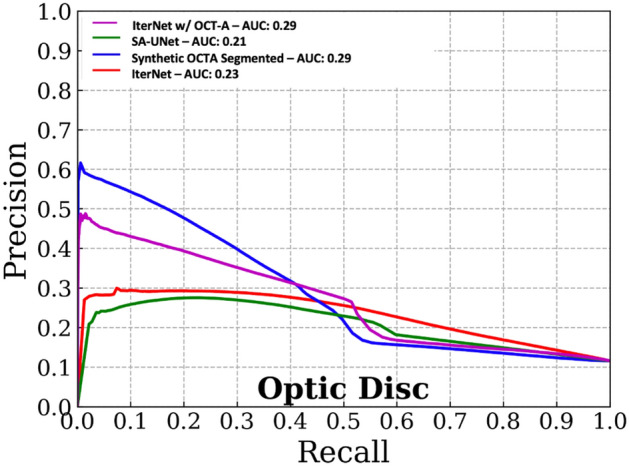
Pixel-wise comparison of our synthetic OCT-A and the two state-of-the-art vessel segmentation algorithms against a binarized OCT-A vasculature on the Macula 3 × 3 mm FOV (Test set).

**Figure 5:**
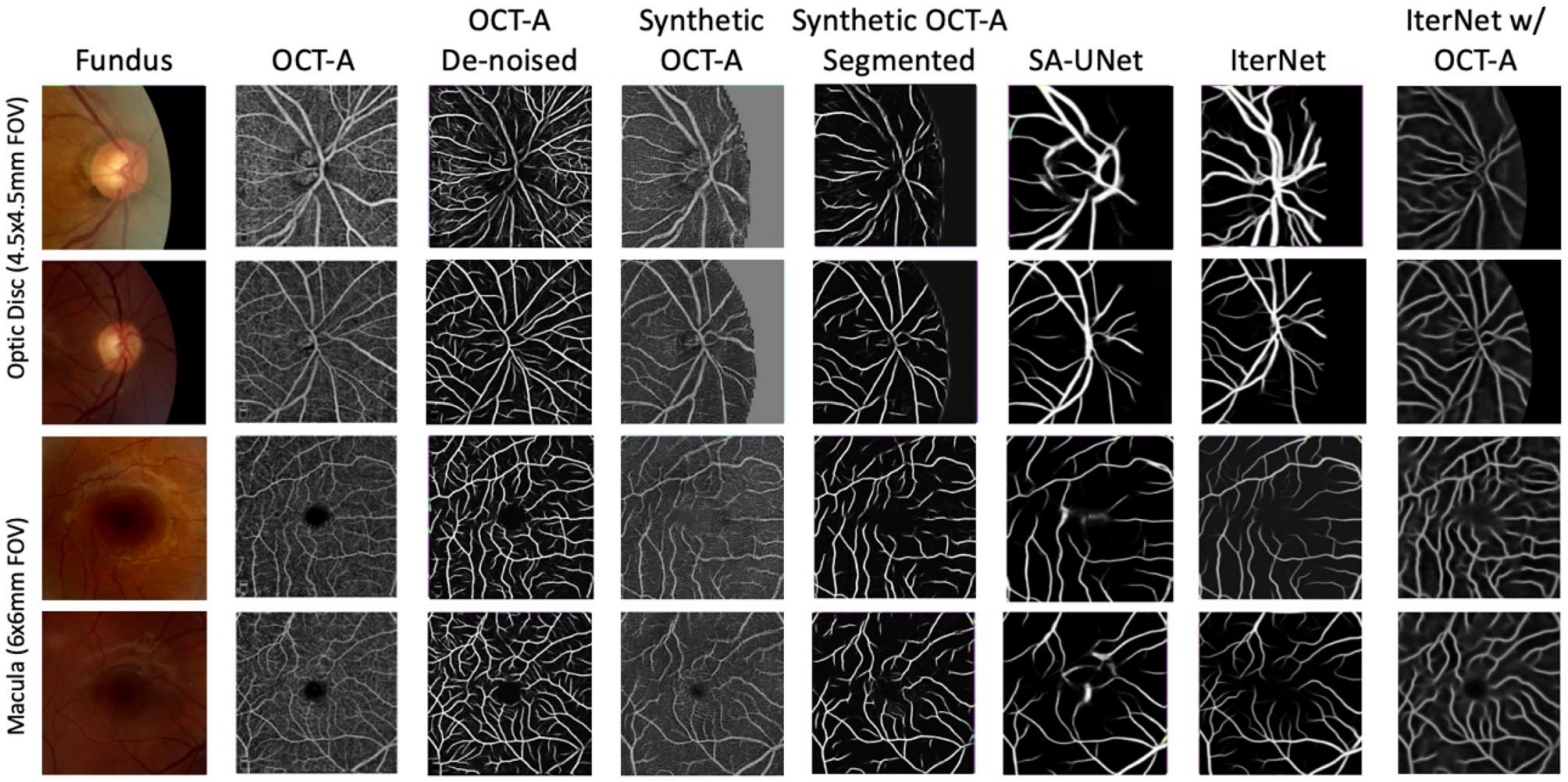
Examples of the output of Synthetic OCT-A and vessel segmentation for the Optic Disc and Macula. Each row represents a set of disc or macula images from subjects belonging to the test set. The “Fundus” column shows the image patch used as input; the “OCT-A” column shows the en face OCT-A used to train the Synthetic OCT-A; the “Synthetic OCT-A” shows the output of the proposed method that is able to highlight vessels and avascular zone; “SA-UNet” and “IterNet” columns show the output of the two state-of-the-art vessel segmentation algorithms used for comparison. The IterNet w/ OCT-A Vessels columns shows the output from the IterNet model that has been trained on vessels segmented from OCT-A.

Figures 3 and 4 show the precision-recall curves for the optic disc and macular regions comparing the synthetic OCT-A and the other vessel segmentations methods. The synthetic OCT-A and the IterNet w/OCT-A better detect retinal blood vessels in the optic disc and are able to find smaller capillaries given high precision despite poor recall. While IterNet is better at capturing macular blood vessels, the overall performance is comparable across the methods given that the obtained values are similar. Figure 5 shows a comparison of the proposed and the other methods for the optic disc and macular regions, including fundus and OCT-A images at the same regions. Synthetic OCT-A and IterNet w/ OCT-A provide a better morphological estimate of the retinal blood vessels compared to IterNet and SA-UNet, both trained on manually segmented vessel maps. We note that the vessels located by the synthetic OCT-A and IterNet w/ OCT-A are tinier and their path more tortuous than those of the vessels in the segmentations produced by the other methods. We attribute these differences to the manual delineation used for training the other segmentation methods.

**Figure 6:**
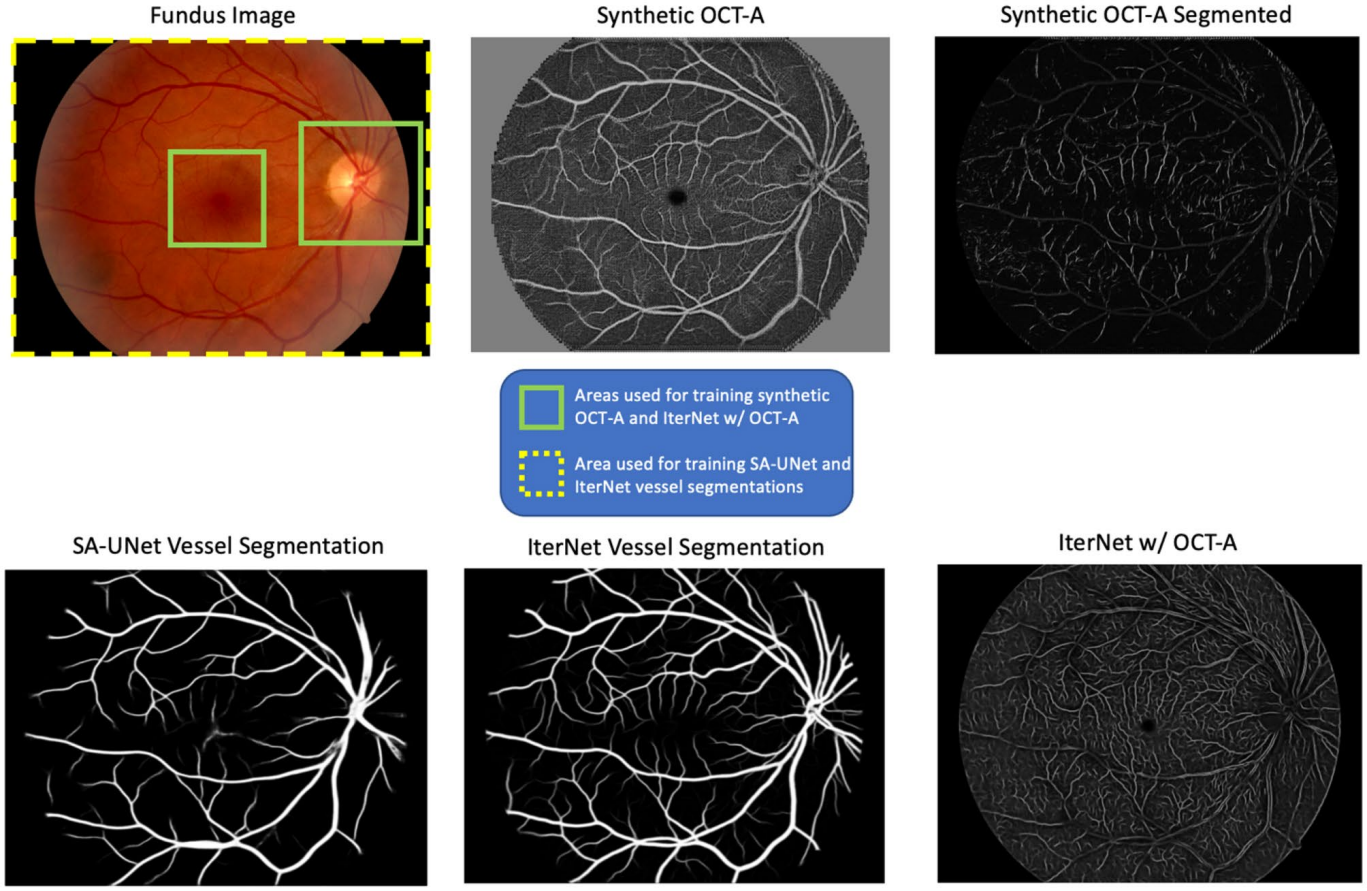
Example of vessel segmentations and synthetic OCT-A from a full 45° FOV fundus image. The synthetic OCT-A is able to generate an estimation of the retinal vasculature and the avascular zone of the full 45° FOV even if it was trained only on smaller FOV from the fundus images and corresponding OCT-A images.

Figure 6 shows vessel segmentation maps for an input fundus photograph. Compared to the IterNet and SA-UNet models that saw manual vessel segmentations for the complete 45° FOV fundus photograph during training, the synthetic OCT-A and IterNet w/ OCT-A models were trained only on samples from the optic disc and macular regions. Nonetheless, both techniques can represent the retinal vasculature outside these areas correctly.

**Figure 7:**
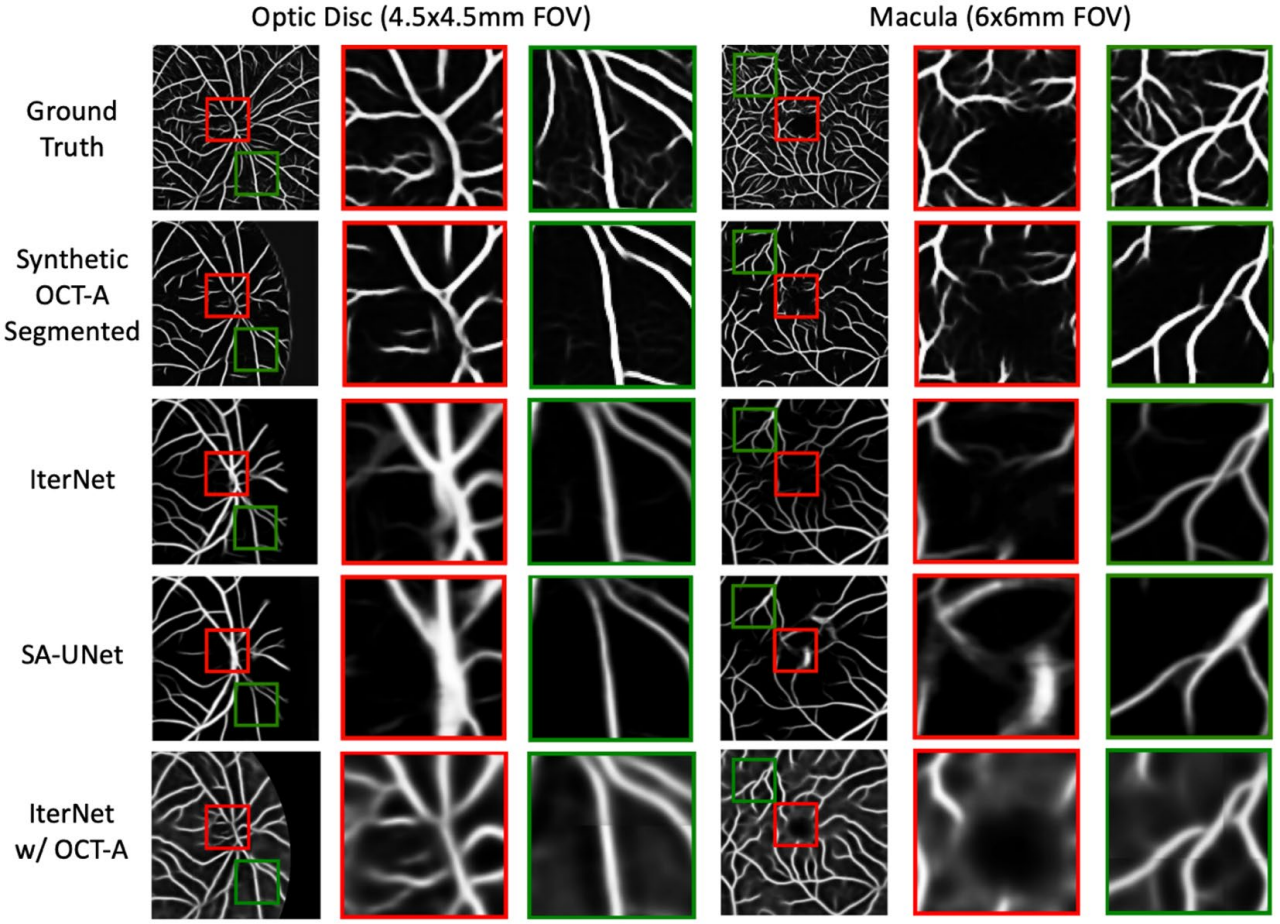
Example of vessel segmentations and synthetic OCT-A for optic disc and macula. The synthetic OCT-A produces an estimation of the vasculature that better captures the morphology and ramifications of small vessels (red squares) but also creates discontinuities in the vessel path (green squares).

Figure 7 shows detailed comparisons between synthetic OCT-A and the other vessel segmentations methods for specific areas of the images. Regions enclosed by red squares depict blood vessels where the synthetic OCT-A yields better representation of vessels given improved branches with less artificial smoothing as opposed to those observed in the IterNet, the IterNet w/OCT-A, and the SA-UNet segmentations.

In addition, regions enclosed by green squares show areas where the synthetic OCT-A was not better or performed poorly compared to the state-of-the-art vessel segmentation approaches, for example, by creating vessel branches nonexistent in the reference OCT-A and also by creating non-contiguous vessels as opposed to the vessels observed in the other methods. All algorithms’ outputs have been made available at https://doi.org/10.5281/zenodo.6476639.

## Discussion

This study introduced an approach to visualize retinal perfusion from fundus images by employing a generative adversarial network to synthesize *en face* 45 degree OCT-A images from local patches of fundus and OCT-A image pairs. OCT-A images provide more precise measures of perfusion, including vessels and other vascular structures, compared to manual segmentation on fundus images, which may face difficulties such as lack of depth information and lower resolution when assessing retinal microvasculature. In fact, the pixels in the OCT-A *en face* are a direct measurement of blood perfusion, with the inevitable addition of noise. As such, the role of a segmentation algorithm in the context of OCT-A can also be considered as a ‘denoising’ process to extract the vascular structures from the accompanying noise found in the raw OCT-A images.

Furthermore, we demonstrated that employing a segmentation network on top of the synthetic OCT-As permits segmenting retinal vessels. While our method does not rely on manual delineations to visualize retinal perfusion, isolating specific structures from the images will require segmentation techniques like the supervised approach by Ma et al.^26^. However, it should be noted that this is not the only available method to obtain a binary vessel mask from OCT-A as other techniques not relying on supervision could be used for post-processing^27^.

Compared to fluorescein angiography, OCT-A does not require the injection of contrast and permits visualizing capillaries more precisely. However, some limitations were observed in the adoption of the OCT-A as a vascular ground truth. First, inter-modality registration was necessary for the model to correctly map pixel-level features between modalities. Also, noise and perfusion artifacts had to be removed to prevent bias in the vessel maps. The former was addressed using a rigid registration algorithm; the latter was performed using an existing vessel segmentation algorithm^26^.

We conducted vessel density analysis to assess how the proposed and established methods (IterNet and SA-UNet) captured retinal vasculature from the fundus images using vessels segmented from OCT-A as the ground truth. Segmenting retinal vessels from fundus photos with the microvascular precision of OCT-A is a difficult task due to the limitations of fundus images compared to OCT-A, such as lacking depth and blood flow information. Compared to the methods trained on manual vessel segmentations from fundus imaging, methods using OCT-A as a target for training (synthetic OCT-A and IterNet w/OCT-A) better represent blood vessels at the optic disc region based on the vessel density and vessel segmentation metrics. IterNet trained on vessel masks delineated from OCT-A was also a better estimator of macular vessel density than all other approaches. This indicates that training on OCT-A vessel information helps obtain more precise delineations of retinal vasculature.

We noticed three relevant qualitative aspects of the vasculature detected by the synthetic OCT-A. First, it tends to detect smaller vessels and non-smooth structures, although it might result in increased false positives at times. Second, vessels within the optic disc region are better represented in the synthetic OCT-A than in manual segmentations, probably due to difficulties encountered by annotators when segmenting the overexposed area of the optic nerve. Finally, when the method is applied beyond the OCT-A’s FOV, it correctly detects vessels in areas of the retina never seen in training data. This is indicative of the good generalizability of the approach.

The generation of synthetic OCT-A leveraged a vanilla U-Net as a backbone. Despite not implementing any of the recent improvements of the basic U-Net, such as attention (SA-UNet)^4^, multi-stages^5^, residual connections^16^, or transformers^28^, the model was able to match and, in some cases, outperform the baselines. Implementing a more complex backend is likely to result in performance improvements as the results using the IterNet w/ OCT-A suggest, yet this would have to be tested in the synthesis of OCT-A.

There are some limitations to our proposed framework. One of them is the Euclidean registration. Due to lens-induced distortions, the rigid inter-modality alignment was not perfect near the image borders. As a result, the adversarial loss would erroneously penalize synthetic images in these areas. This problem was deemed marginal and not worth addressing with nonrigid registration. Another limitation is that our model does not create a vessel segmentation but rather an estimation of the *en face* OCT-A corresponding to a fundus image. Further postprocessing is required to extract vessels accurately from the synthetic OCT-A image. However, this could also be considered advantageous, as there are other potential uses of synthetic OCT-A beyond vessel segmentation. One example is the estimation of the avascular zone, currently only conducted with OCT-A or other angiography techniques. Another aspect to consider is that SA-UNet and IterNet were tested using the network weights provided by the authors, which were trained using fundus images different from the ones used to train our synthetic OCT-A approach. This is likely to disadvantage our approach, as SA-UNet and IterNet were trained on much larger image datasets. To conduct a fair evaluation, we trained a version of IterNet on vessels segmented from OCT-A. Our results showed that this approach attained improved vessel segmentation for both macula and optic disc areas. This further reinforces our hypothesis, suggesting an added benefit in training segmentation models using information extracted from OCT-A. Finally, refinement of our algorithm will require additional pairs of fundus and OCT-A images, compared to our approach, creating a dataset with manually delineated vessel segmentations will be the most cost-effective approach in some settings. This study introduced a strategy to map fundus images to an *en face* OCT-A image, creating precise vascular segmentations with less effort as manual delineation is unnecessary in our methodology. Being the first effort of its kind, it is likely that this synthetic OCT-A algorithm could be further improved. Therefore, as a contribution to the ongoing efforts by the scientific community and to motivate interest in this line of research, we make our data available for non-commercial research at 10.5281/zenodo.6476639.

While limited information is observed in fundus images, OCT-A permits visualizing additional vascular information; however, due to its limited availability it is not feasible for many applications. As suggested by our results, synthetic OCT-A permits extracting detailed vascular information from fundus images alone which has important implications for screening pathologies and triaging patients.

This paper compared and contrasted methodologies for estimating retinal vasculature using enface OCT-A as training target. In addition to standard segmentation trained directly with vessel segmented with OCT-A, we proposed a modality transformation model to obtain a synthetic version of the OCT-A modality from a corresponding fundus photograph. Quantitative and qualitative evaluations against state-of-the-art vessel segmentation models demonstrated how OCT-A could be used as an alternative to manually delineated vascular maps simplifying the creation of vascular labels for retinal datasets.

Future work will focus on experimenting with enhancements of the synthesis model building blocks (e.g., attention, multi-staging) and on combining the OCT-A synthesis with a classic vessel segmentation approach to obtain an improved version of the synthetic OCT-A. In addition, we will experiment with uses of the synthetic OCT-A beyond vessel segmentation, such as the estimation of the avascular zone.

## Data Availability

Source images and output from the algorithm presented are available for non-commercial research purposes at https://zenodo.org/record/6476638.
