# Author Correction: Synthetic OCT-A blood vessel maps using fundus images and generative adversarial networks

**DOI:** 10.1038/s41598-023-43285-6

**Published:** 2023-09-27

**Authors:** Ivan Coronado, Samiksha Pachade, Emanuele Trucco, Rania Abdelkhaleq, Juntao Yan, Sergio Salazar‑Marioni, Amanda Jagolino‑Cole, Mozhdeh Bahrainian, Roomasa Channa, Sunil A. Sheth, Luca Giancardo

**Affiliations:** 1 McWilliams School of Biomedical Informatics, University of Texas Health Science Center at Houston, Houston, TX USA; 2 VAMPIRE project, School of Science and Engineering (Computing), University of Dundee, Dundee, Scotland, UK; 3 McGovern Medical School, University of Texas Health Science Center at Houston, Houston, TX USA; 4 Department of Ophthalmology and Visual Sciences, University of Wisconsin-Madison, Madison, WI USA

Correction to: *Scientific Reports* 10.1038/s41598-023-42062-9, published online 15 September 2023

The original version of the Article contained an error in the Author Information statement.

“These authors contributed equally: Ivan Coronado, Samiksha Pachade, Roomasa Channa, Sunil A. Sheth and Luca Giancardo.”

now reads:

“These authors contributed equally: Ivan Coronado and Samiksha Pachade.”

“These authors jointly supervised this work: Roomasa Channa, Sunil A. Sheth and Luca Giancardo.”

The original Article has been corrected.

